# Harmonic Sierpinski Gasket and Applications

**DOI:** 10.3390/e20090714

**Published:** 2018-09-17

**Authors:** Emanuel Guariglia

**Affiliations:** 1 Department of Mathematics and Applications “R.Caccioppoli”, University of Naples Federico II, 80126 Naples, Italy; emanuel.guariglia@gmail.com; 2 Department of Mathematics, University of Trento, 38123 Trento, Italy

**Keywords:** antenna, harmonic function, harmonic Sierpinski gasket, homeomorphism, Rényi entropy, self-affine fractal, Sierpinski gasket

## Abstract

The aim of this paper is to investigate the generalization of the Sierpinski gasket through the harmonic metric. In particular, this work presents an antenna based on such a generalization. In fact, the harmonic Sierpinski gasket is used as a geometric configuration of small antennas. As with fractal antennas and Rényi entropy, their performance is characterized by the associated entropy that is studied and discussed here.

## 1. Introduction

In recent years, entropic theories have given rise to considerable interest in mathematics, physics, engineering and applied science. On the other side, chaotic motions and attractors are often modeled by iterative maps, that is fundamental methods of fractal geometry. Chaos, entropy and fractals have drawn the interest of many researchers due to their mathematical modeling ability to solve a wide variety of real problems. In particular, Kolmogorov generalized the concept of entropy in order to define a fundamental measure for chaotic evolution [1]. This definition allows the classification of dynamical systems as regular, chaotic and purely random.

Fractal sets are characterized by their self-similarity property, that is each part of the set has the same or approximate shape of the whole set. However, fractal sets as each mathematical abstraction are unable to provide a model for real-world applications. This issue can be overcome with the introduction of the notion of pre-fractals, which are fractals built with a finite number of iterations. In recent times, pre-fractal modeling has provided huge versatility in engineering and applied science [2,3]. In particular, pre-fractals are used to design the geometric configuration of small antennas called fractal antennas. A pre-fractal structure makes these antennas multiband with efficient miniaturization. In fact, fractal geometry entails two main advantages supplied by self-similarity and space-filling properties [2]. In the class of fractal antennas, the Sierpinski gasket antenna plays a fundamental role in research due to its high broadband performance. In fact, it is widely used in wireless communication systems (UMTS, WLAN, etc.), spatial communication (RF MEMS probes) and ANN [4,5]. Taking into account the link between the Rényi entropy and the Rényi dimension [6], the entropy of fractal antennas can easily be defined and computed [1]. Consequently, the entropy of such antennas results in a direct link with their geometric configuration representing an antenna performance parameter.

In topological terms, the (Euclidean) Sierpinski gasket is a fractal set, which cannot be seen as a smooth or topological manifold. Nevertheless, it turns out that this fractal owns a natural metric structure induced by the Euclidean metric in $R^{2}$. In [7,8], Kigami generalized the Sierpinski gasket through the theory of harmonic functions. The new metric space, homeomorphic to the Sierpinski gasket, is called the harmonic gasket or the Sierpinski gasket in harmonic metric (cf. [9,10,11] and the references given there). In this paper, it will simply be called the harmonic Sierpinski gasket for brevity and to avoid any confusion. The harmonic generalization of the ordinary Sierpinski gasket immediately attracted considerable attention due to its applications in probability theory and harmonic analysis [7,12,13,14,15]. In particular, this paper shows the main properties of the harmonic Sierpinski gasket together with an application to antenna design.

The remainder of this paper is organized as follows. Section 2 presents some preliminaries on fractal geometry and on Rényi entropy. Section 3 is intended to motivate our investigation of the harmonic Sierpinski gasket. Section 4 is devoted to the study of the associated antenna. Finally, Section 5 outlines the main results of this paper, open problems and possible future developments.

## 2. Preliminaries

This section contains some general remarks on fractal geometry and Rényi entropy. As a result, the next sections will be rendered as self-contained as possible in order to facilitate access to the individual topics. Excepting the notation herein introduced, we refer the reader to [1].

### 2.1. Fractal Geometry and Sierpinski Gasket

Fractal sets are mainly described by the fractal dimension, an index of complexity introduced by Mandelbrot and based on his 1967 paper on fractional dimensions ([2], p. xxvii). Fractal sets with the same fractal dimension can be extremely different. As a result, the fractal dimension does not uniquely characterize self-similar sets. Nevertheless, it remains the most important parameter in fractal modeling. Unlike topological dimensions, the fractal dimension is a non-integer value, which indicates how a set fills the space. This lack makes the traditional geometries ill-suited for the characterization of several sets, currently known as fractals. The fractal dimension has been studied extensively in a variety of literature [2]. The fractal dimension can be theoretically defined and empirically measured in many ways. However, the only introduction of the Besicovitch measure has allowed the definition of dimension for extremely irregular sets. This value, called the Hausdorff–Besicovitch dimension (Hausdorff dimension for short), raised the fractal modeling to the role of mathematical theory. In fact, Mandelbrot defined “a fractal as a set for which the Hausdorff-Besicovitch dimension strictly exceeds the topological dimension”. Despite this central role in fractal geometry, it can turn out to be unsuitable for real-world applications. However, there are other definitions in widespread use due to both their ease in mathematical computation and empirical estimation. It is not our purpose to study in detail the Hausdorff–Besicovitch dimension ([2], Chapter 2), and we restrict our attention to another definition of fractal dimension called the box-counting dimension. It has several applications in mathematics, science and engineering and is reported ([2], p. 41) below.

**Definition** **1** (Box-counting dimension)**.** *Let A be a non-empty bounded subset of $R^{n}$, and let $N_{\delta }\left(A\right)$ be the smallest number of δ-boxes needed to cover A. The lower and upper box-counting dimensions of A, denoted respectively by ${\underline{\mathrm{Dim}}}_{B}\left(A\right)$ and ${\bar{\mathrm{Dim}}}_{B}\left(A\right)$, are defined as follows*

$$\begin{array}{cc} {\underline{\mathrm{Dim}}}_{B}\left(A\right) & =\underset{\delta \to 0}{lim inf}\frac{\log N_{\delta }\left(A\right)}{\log(1/\delta )}\;,\\ {\bar{\mathrm{Dim}}}_{B}\left(A\right) & =\underset{\delta \to 0}{lim sup}\frac{\log N_{\delta }\left(A\right)}{\log(1/\delta )}\;. \end{array}$$

*If ${\underline{\mathrm{Dim}}}_{B}\left(A\right)={\bar{\mathrm{Dim}}}_{B}\left(A\right)$, this common value is called the box-counting dimension of A. Thus, the box-counting dimension of A is given by*

(1) $${\mathrm{Dim}}_{B}\left(A\right):=\lim_{\delta \to 0}\frac{\log N_{\delta }\left(A\right)}{\log(1/\delta )}\;,$$

*if the limit* (1) *exists.*

Therefore, the box-counting dimension of *A* is defined by the minimum number of δ-boxes (or any other equivalent set of side δ) that cover the set *A* lying in an evenly-spaced grid. The box-counting dimension can also differ from the Hausdorff–Besicovitch dimension. In fact, under the hypothesis and notation of Definition 1, it is easy to show ([2], p. 46) that $0\leq {\mathrm{Dim}}_{H}\left(A\right)\leq {\mathrm{Dim}}_{B}\left(A\right)$. Hence, the box-counting dimension is an upper estimation of the Hausdorff–Besicovitch dimension. In fractal geometry, fractal sets can be built by IFS. Based on the self-similarity, it has been at the heart of fractal geometry almost from its origins.

**Definition** **2** (IFS)***.** Let D be a closed subset of $R^{n}$. A family of contractive maps $F_{1},\ldots ,F_{m}$ on D is called an iterated function system (IFS). By IFS theory ([2], p. 123), a non-empty compact subset A of D is an attractor of the IFS if*

(2) $$A:=\overset{m}{\underset{i=1}{⋃}}F_{i}\left(A\right)\;.$$

*Furthermore, A is called a self–affine fractal (or self–affine set) if the $F_{i}$ are affine transformations on $R^{n}$, that is*

$$F_{i}x=T_{i}x+d_{i}\;,\;\left(1\leq i\leq m\right)$$

*where $T_{i}$ are linear transformations on $R^{n}$ and $d_{i}\in R^{n}$ are translation vectors.*

**Figure 1:**
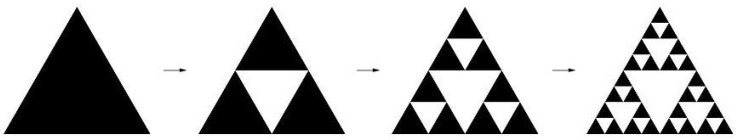
Construction of the Sierpinski gasket [1].

In Definition 2, often, $D=R^{n}$. The fundamental property of an IFS, pioneered by Hutchinson [16], is that it determines a unique attractor. In the special case when $F_{i}$ are all similarities, *A* is self-similar. For our purposes, the class of self-affine fractals is of particular interest. Self-affine fractals are scaled by different amounts in different directions. In the case of self-similarity, the fractal is scaled by the same amount in all directions. Self-affine fractals are generally fractal, and it is natural to investigate their applications in pure and applied mathematics ([2], Chapter 9). In particular, the self-affinity of the harmonic Sierpinski gasket is shown in Section 3. The IFS plays a central role in fractal geometry given that fractal sets are made up of parts that are similar, in some way, to the whole. Each self-similar step can be seen as a contraction, that is the construction of the fractal set comes over from the iteration of the Banach contraction mapping theorem. Consequently, many fractal sets can be characterized by contraction factors $c_{1},\ldots ,c_{m}$. However, a detailed description of the IFS exceeds the scope of this paper. In addition to characterizing the construction of fractal sets, IFS often leads to a simple way to compute dimensions. For several fractal sets, this value can be determined in a much easier way. For instance, the Moran–Hutchinson theorem ([2], pp. 130–132) enables us to find the dimension of many self-similar fractals. This theorem states that

$$\sum_{k=1}^{m}c_{k}^{s}=1\;,$$

where *s* is the fractal dimension. As a worthwhile example, we can consider the Sierpinski gasket. It is simply denoted by *K*. This fractal is built from an equilateral triangle by the removal of inverted equilateral triangles, as shown in Figure 1. Clearly, $c_{1}=c_{2}=c_{3}$, which gives

$$3{\left(\frac{1}{2}\right)}^{s}=1\;.$$

Therefore, the fractal dimension of *K* is given by

$$s=\frac{\log3}{\log2}\approx 1.584962\;.$$

In Figure 1, the basic version of *K* is shown. In fact, equivalent definitions and generalizations of this fractal are widely present in the current literature. In particular, the shape and topological model can be adapted to the application sought. As a result, *K* has a broad range of applications (especially in antenna theory). As mentioned in the Introduction, all the fractal applications in science and engineering are based on pre-fractal models. Additionally, the main advantages vanish after a few iterations. For fractal antennas, such a number is almost never greater than six [1].

### 2.2. Rényi Entropy

The concept of entropy arises in thermodynamics and statistical physics. Shannon adapted such a concept to signal theory, laying the foundations of information theory and modern telecommunications [17]. A few years later, Rényi provided a first generalization of the Shannon entropy [1,18,19]. It is currently known as Rényi entropy and is defined below.

**Definition** **3** (Rényi entropy)**.** *Let X be a discrete random variable and let $\alpha \in R_{\geq 0}$. The Rényi entropy of order α is given by*

(3) $$H_{\alpha }\left(X\right):=\frac{1}{1-\alpha }\log_{b}\sum_{i=1}^{N}p_{i}^{\alpha }\;,$$

*where $p_{i}$ is the probability of the event $\left\{X=x_{i}\right\}$.*

In Definition 3, the most common values for *b* are b=2 and b=e. Of course, it is necessary that α≠1 to avoid beingzero. Let us show that the $H_{\alpha }$ is well defined. L’Hôpital’s rule entails that

$$\frac{1}{1-\alpha }\log_{b}\sum_{i=1}^{N}p_{i}^{\alpha }\overset{\;\;\alpha \to 1\;\;}{\to }H\left(X\right)\;,$$

that is, $H_{1}$ is mandatorily defined as the Shannon entropy. Consequently, Rényi entropy is a generalization of the Shannon entropy; see [20] for more details. Furthermore, from Definition 3, it follows that $H_{\alpha }$ characterizes the randomness of complex systems [6].

In fractal geometry, the Hausdorff–Besicovitch dimension does not provide a constructive method to estimate the fractal dimension. Accordingly, other fractal parameters (box-counting dimension, lacunarity, etc.) have been introduced over time in order to solve these potential problems [2,21]. In particular, the different definitions of fractal dimension allow fractal modeling to be suitable for real-world applications. For our purposes, the fractality will be estimated via a generalized fractal parameter called the Rényi dimension [6].

**Definition** **4** (Rényi dimension)**.** *Under the same hypotheses as in Definitions 3, the Rényi dimension of order α is given by*

$$D_{\alpha }\left(X\right):=\frac{1}{\alpha -1}\lim_{\delta \to 0}\frac{\log_{b}\sum_{i=1}^{N}p_{i}^{\alpha }}{\log_{b}\delta }\;,$$

*where N=N(δ) is the total number of δ-boxes with $p_{i}>0$.*

As in Definition 3, it is understood that α≠1. In fact, applying L’Hôpital’s rule

$$\frac{1}{\alpha -1}\lim_{\delta \to 0}\frac{\log_{b}\sum_{i=1}^{N}p_{i}^{\alpha }}{\log_{b}\delta }\overset{\;\;\alpha \to 1\;\;}{\to }\lim_{\delta \to 0}\frac{\sum_{i=1}^{N}p_{i}\log_{b}p_{i}}{\log_{b}\delta }\;.$$

Hence, the above definition of the Rényi dimension makes sense. It directly follows that

(4) $$D_{\alpha }=-\lim_{\delta \to 0}\frac{H_{\alpha }}{\log_{b}\delta }\;,$$

which justifies the appellation of the Rényi dimension. The parameter $D_{\alpha }$ is also known as the generalized fractional dimension since

$$D_{0}=\lim_{\delta \to 0}\frac{\log_{b}N}{\log_{b}\left(\frac{1}{\delta }\right)}\;,$$

that is the box-counting dimension. The cases α=1 and α=2 are of special interest in chaos theory. In fact, $D_{1}$ and $D_{2}$, called information dimension and correlation dimension, respectively, are used to describe the behavior of chaotic attractors. As a result, the Rényi dimension characterizes fractality, randomness and chaoticness. The main difficulty is that $D_{\alpha }$ cannot be directly computed. Nevertheless, for any fixed δ>0 small enough, we get

(5) $$H_{\alpha }=-D_{\alpha }\;\log_{b}\delta \;,$$

**Figure 2:**
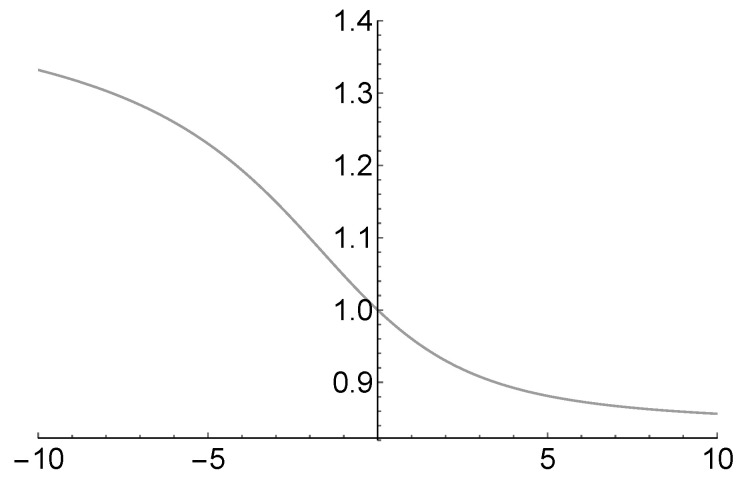
Rényi dimension $D_{\alpha }$ for N=3, δ=1/3, $p_{1}=1/5$, $p_{2}=p_{3}=2/5$ and α∈[−10,10].

Approximation (5) allows the entropy of a region to be computed from the three spatial coordinates [22]. Therefore, the entropy of fractal antennas can be defined by the introduction of $D_{\alpha }$. In particular, the entropy of the Sierpinski gasket antenna is computed and discussed in [1]. The entropy described by Formula (5) depends on the computation of the Rényi dimension. It describes the so-called multifractality, which is the generalization of the concept of fractality in which different scaling factors occur with different probabilities. In particular, for identical scaling factors, the dependence of $D_{\alpha }$ on α can be easily represented as in Figure 2.

Figure 2 shows that $D_{\alpha }$ is a nonincreasing function of α on [−10,10]. This property holds on the whole real line (see [6] for more details). The numerical computation of $D_{\alpha }$ is based on the auxiliary function $\tau \left(\alpha \right)=\left(\alpha -1\right)D_{\alpha }$ and is sufficient for our purposes. However, this procedure allows us to determine the multifractal spectrum $f_{\alpha }$ through the Hölder exponent [23].

## 3. Harmonic Sierpinski Gasket

The construction of *K* enables one to define a new structure on the gasket. This generalization of *K* can be defined by homeomorphism theory in terms of harmonic functions. In particular, approximations as in Figure 1 can be characterized by a harmonic metric. The construction does not imply any additional issue. In fact, harmonic functions defined in terms of graphs are continuous in the Euclidean topology of the gasket. *K* is the attractor of the IFS given by

(6) $$F_{i}x=\frac{1}{2}\left(x-p_{i}\right)+p_{i}\;,\;\left(\mathrm{for}\;\;i=1,2,3\right)$$

in which $p_{i}$ are the vertices of an equilateral triangle. Although the space of the harmonic functions is three-dimensional, such functions can be reduced to one variable by composing on the right with an isometry of *K* and on the left with an affine mapping [24]. The IFS (6) can be generalized to a regular three-simplex as in [9]. However, *K* is the unique nonempty compact subset of $R^{2}$ such that $K={⋃}_{i=1}^{3}F_{i}\left(K\right)$.

**Definition** **5** (Decomposition in m-cells)**.** *For any m≥1, let w be the multi-index defined by $w=(w_{1},w_{2},\ldots ,w_{m})$ such that $w_{i}=\left\{1,2,3\right\}$ for i=1,…,m and $F_{w}$ be the IFS defined by $F_{w}=F_{w_{1}}\circ \cdots \circ F_{w_{m}}$. The decomposition of K into m-cells is given by*

(7) $$K:=\underset{|w|=m}{⋃}F_{w}\left(K\right)\;.$$

In Definition 5, for the simplicity of notation, $F_{w}\left(K\right)$ is often denoted by $K_{w}$ since the *m*-cells depend on *w*. First and foremost, we need to show how the theory of harmonic functions can be generalized on *K*. In order to get such a definition, we have to provide the concept of energy, which for harmonic functions is easily explained. For this purpose, some basic remarks on the graph approximation of *K* and their associated vertices are given [24]. In particular, the concept of the multi-index enables us to describe the points of *K* by words, whose elements belong to T={1,2,3}. Let $\Sigma =T^{N}$, $W_{0}=\left\{\emptyset \right\}$ and $W_{m}=T^{m}$ for m>0. Hence, for any m≥0, the set $W_{m}$ contains all the words of length *m*. Under these assumptions, the vertices of *K* are given by $V_{*}={⋃}_{m\geq 0}V_{m}$ with $V_{0}=\left\{p_{1},p_{2},p_{3}\right\}$ and

(8) $$V_{m}=\underset{w\in W_{m}}{⋃}F_{w}\left(V_{0}\right)\;.$$

The Sierpinski gasket *K* can be seen as the limit of the graphs $\Gamma_{m}$ with vertices $V_{m}$ and an appropriate edge relation. Let $\Gamma_{0}$ be the complete graph of $V_{0}$. The relation x≅y if and only if *x* and *y* are neighbors in $\Gamma_{m}$ can be inductively defined by Formula (8). It provides the desired link between *K* and $V_{*}$. Note that $V_{m-1}\subseteq V_{m}$. Hence, $V_{m}\backslash V_{0}$ consists of all non-boundary vertices in $\Gamma_{m}$. It is immediate that all these vertices have always four neighbors in $V_{m}$ (see Figure 1). Therefore, $\Gamma_{m}$, called the graph cell, is given [9] by

$$\Gamma_{m}:=\left\{\begin{array}{cc} V_{0}\;,\; & |w|=0\;,\\ F_{w}\left(V_{0}\right)\;,\; & |w|>0\;. \end{array}\right.$$

**Definition** **6** (Energy)**.** *The graph energy form on $\Gamma_{m}$, $E_{m}\left(u,v\right)$, is given by*

$$E_{m}\left(u,v\right):={\left(\frac{5}{3}\right)}^{m}\sum_{x≅y:x,y\in V_{m}}\left(u(x)-u(y)\right)\left(v(x)-v(y)\right)\;,$$

*where $V_{m}$ is the set of vertices in $\Gamma_{m}$ and the relation x≅y is defined above. The energy, E(u), on K is given by*

$$E\left(u\right)=\lim_{m\to \infty }E_{m}\left(u,u\right)\;.$$

The theory of harmonic functions on *K* is part of a more general theory based on the Laplacian Δ. According to this theory, *u* is harmonic if and only if Δu=0. However, the harmonic condition Δu=0 can be replaced by an energy minimization condition [12,25,26,27,28].

**Definition** **7** (Harmonic extension)**.** *Let u be defined on $V_{0}$. The unique extension of u from $V_{0}$ to $V_{m+1}$, denoted by $\hat{u}$, is called the harmonic extension of u if it minimizes the energy $E_{m+1}$ [9] by*

$$E_{0}\left(u\right)={\left(\frac{5}{3}\right)}^{m}E_{m+1}\left(\hat{u}\right)\;.$$

Definition 7 assures that given values of a function *u* on $V_{0}$, it can be uniquely extended to $V_{m}$ for any *m*. Therefore, *u* can be extended to $V_{*}$. The function *u* is called a harmonic function on *K*. Clearly, any harmonic function *u* is determined uniquely by ${u|}_{V_{0}}$. We can now proceed with the construction on *K*, which can be seen as a space to be geometrized [7]. In fact, the metric harmonic makes *K* a geometric space called the harmonic (Sierpinski) gasket. As a result, the harmonic gasket is introduced by the space of harmonic functions H. The last step in the construction of $K_{H}$ is given using these harmonic functions as a coordinate chart for *K* in the subspace $M_{0}:=\left\{\left(x,y,z\right)\in R^{3}\;:\;x+y+z=0\right\}$. Kigami [7] introduced the map

(9) $$\Phi :K\to M_{0}\;,$$

by

$$\Phi \left(x\right)=\frac{1}{\sqrt{2}}\left(\left(\begin{array}{c} h_{1}\left(x\right)\\ h_{2}\left(x\right)\\ h_{3}\left(x\right) \end{array}\right)-\frac{1}{3}\left(\begin{array}{c} 1\\ 1\\ 1 \end{array}\right)\right)\;.$$

**Figure 3:**
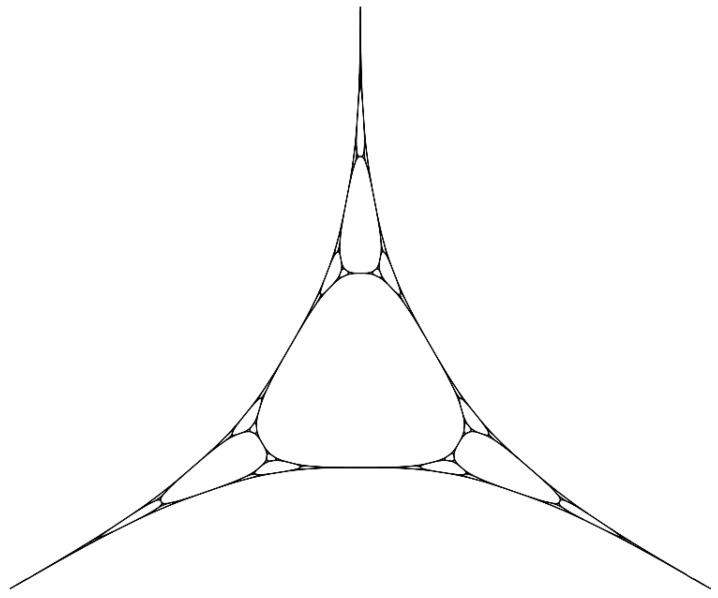
The harmonic Sierpinski gasket $K_{H}$ as a homeomorphic image of *K*.

In the map above, $h_{i}\left(p_{j}\right)=\delta_{ij}$ for i,j=1,2,3 and $p_{j}\in V_{0}$. In order to clarify the role played by such functions, without loss of generality, we can identify H with $R^{3}$. As already mentioned, a harmonic functions depends uniquely on its value on $V_{0}$. Therefore, $\left\{h_{1}=\left(1,0,0\right),\;h_{2}=\left(0,1,0\right),\;h_{3}=\left(0,0,1\right)\right\}$ is a basis for H. The map Φ is a homeomorphism onto its image. The action of the map Φ on *K* is shown in Figure 3. Therefore, K≃Φ(K) leads to define the harmonic Sierpinski gasket, denoted by $K_{H}$. This generalization of *K* is also called the harmonic gasket (for brevity) or Sierpinski gasket in the harmonic metric. It is not a fractal set. Nevertheless, the following theorem provides a natural and intrinsic characterization of $K_{H}$.

**Theorem** **1.** *The harmonic Sierpinski gasket $K_{H}$ is a self-affine fractal in $R^{2}$.*

**Proof.**  The harmonic Sierpinski gasket is defined by the homeomorphism Φ, which preserves compactness. Therefore, $K_{H}$ is a compact subset of $M_{0}$. The proof consists of the construction of the affinities $H_{i}:M_{0}\to M_{0}$ as in Definition 2. Let $P:R^{3}\to M_{0}$ be the orthogonal projection defined by

$$q_{i}=\frac{P\left(e_{i}\right)}{\sqrt{2}}\;,\;\left(\mathrm{for}\;\;i=1,2,3\right)$$

in which $\left\{e_{1},e_{2},e_{3}\right\}$ is the natural basis of $R^{3}$. Furthermore, for any i=1,2,3, let $J_{i}:M_{0}\to M_{0}$ be the maps defined by

$$J_{i}\left(q_{i}\right)=\frac{3}{5}q_{i}\;\mathrm{and}\;J_{i}\left(g_{i}\right)=\frac{g_{i}}{5}\;,$$

where $g_{i}$ are such that $\left\{g_{i},\frac{q_{i}}{|q_{i}|}\right\}$ is an orthonormal basis of $M_{0}$. The affinities sought are given by $H_{i}\left(x\right)=J_{i}\left(x-q_{i}\right)+q_{i}$ for any i=1,2,3. Therefore, $K_{H}$ is given by $K_{H}={⋃}_{i=1}^{3}H_{i}\left(K_{H}\right)$, which completes the proof.  ☐

Note that the contractions $H_{i}$ are clearly linked to the contractions $F_{i}$ used to build *K* through the homeomorphism (9). In fact, Φ commutes with such contractions, that is $\Phi \circ F_{i}=H_{i}\circ \Phi$ for i=1,2,3. As a result, $K_{H}$ can also be built via Φ from the contractions $F_{i}$. The harmonic Sierpinski gasket shows the importance of the harmonic analysis in fractal geometry. In the long term, further analytic developments of the harmonic Sierpinski gasket might be of independent interest in pure and applied mathematics(see for instance [29]).

## 4. An Application in Antenna Theory

Fractal antennas have become very popular since 1988. In the current literature, this year is widely recognized as the year of their birth, when Cohen published a paper about this new type of antenna [1]. The pre-fractal model entails both self-similarity and space-filling, which make fractal antennas suitable for military, space and multiband application. Hence, fractal antennas have a large effective length due to their pre-fractal contour. In addition, the importance of fractal antennas is borne out from their application in metamaterials. The class of metamaterials called fractal metamaterials allows making invisible a variety of objects as satellites, spacecraft and even people [30]. However, the fractal geometry does not uniquely translate into the electromagnetic behavior of the antenna. In fact, despite all these advantages, non-fractal antennas can reach or exceed the performance of their fractal counterparts. This is in accordance with antenna theory. In 1999, a characterization to make antennas’ frequency invariant was established. This is now known as the HCR principle and describes necessary and sufficient conditions for all frequency independent antennas [31]. According to this principle, the self-similarity is the main requirement for the frequency independence, together with origin symmetry. Therefore, non-fractal antennas that satisfy the HCR principle can also be frequency independent, providing similar performance to those with pre-fractal contour [1].

### Entropy of Self-Affine Fractal Antennas

Theorem 1 allows an application of the harmonic Sierpinski gasket in antenna theory. For simplicity, this will be called the harmonic gasket antenna. The harmonic Sierpinski gasket can be used as a geometric configuration in antenna design. Clearly, the antenna design will use a pre-fractal version of the harmonic Sierpinski gasket, that is the IFS is built with a finite number of iterations. As already mentioned in Section 2, a self-affine fractal is given by contractions that scale the set by different factors, horizontally and vertically. Accordingly, this self-affine geometric configuration can provide further flexibility in design. This property is due to the appropriate management of the two different scale factors, which enables one to space resonances by different factors. In self-affine antenna design, additional degrees of freedom are often introduced by perturbing the antenna shape and varying each segment length and thickness, in order to both increase the chaoticness of the structure and fit more challenging project requirements. In [1], the Rényi entropy of the Sierpinski gasket antenna is defined and discussed by Formula (5). For pre-fractal sets, the Rényi dimension can be numerically computed. In addition, other parameters (fractal spectrum, Hölder exponent, etc.) are easily derived with similar numerical techniques. Taking into account that the concept of multifractality can be extended to self-affine fractals [32], the entropy of the harmonic gasket antenna can easily be computed. For more details, we refer the reader to [18,19].

There is an easier method to compute the entropy of some self-affine fractal antennas based on the additivity of the Rényi entropy. Suppose that the self-affine fractal can be partitioned into self-similar fractals. Once the self-affine fractal structure is chosen, the numerical computation reduces to that described for the Sierpinski gasket antenna [1]. Hence, the entropy of these self-affine fractal antennas is given by

(10) $$H=\sum_{i=1}^{m}H_{\alpha_{i}}\;,$$

where $H_{\alpha_{i}}$ is the Rényi entropy of the *i*-th pre-fractal and *m* is the total number of pre-fractal subsets that partition the antenna. This method reduces the computational cost. In fact, the entropy (10) is the sum of all the Rényi entropies associated with each self-similar subset. As already mentioned in Section 2, the main advantages of the pre-fractal configuration in antenna design vanish after a few iterations (typically five or six iterations). Therefore, the entropy (10) is the sum of no more than six Rényi entropies. Formulas (5) and (10) entail that the entropy sought is characterized by the Rényi dimension of each self-similar subset. Accordingly, the method described above is nothing more than a simple generalization of that introduced in [1] for the Sierpinski gasket antenna.

For example, let us consider the Rényi dimension in Figure 2. The associated model consists of three independent sets, characterized by the probabilities $p_{1}=1/5$ and $p_{2}=p_{3}=2/5$. In general, the performance of the self-affine antenna can be expressed in terms of multifractal analysis. For any fixed δ>0 small enough, Formula (5) holds where

$$D_{\alpha }=\frac{1}{\alpha -1}\frac{\log_{b}\left(1+2^{\alpha +1}\right)+\alpha \log_{b}p_{1}}{\log_{b}\delta }\;.$$

The multifractal spectrum $f_{\alpha }$ is given [23] by

$$\left(\alpha -1\right)D_{\alpha }=\alpha H_{\alpha }-f_{\alpha }\;,$$

in which $H_{\alpha }$ is the Hölder exponent. A simple computation gives

$$H_{\alpha }=\frac{1}{\log_{b}\delta }\left(\frac{2^{\alpha +1}\log2}{1+2^{\alpha +1}}+\log p_{1}\right)\;,$$

therefore

(11) $$f_{\alpha }=\frac{1}{\log_{b}\delta }\left(\frac{d}{d\alpha }\log\left(1+2^{\alpha +1}\right)-\log\left(1+2^{\alpha +1}\right)\right)\;.$$

Formula (11) does not depend on $p_{1}$. Of course, $D_{\alpha }$, $H_{\alpha }$ and $f_{\alpha }$ cannot be solved explicitly. Their numerical computation, as in Figure 2, allows one to achieve information on the performance of each self-affine antenna. Whenever Equality (10) holds, the analysis of the self-affine antenna is simplified. In fact, it reduces to the computation of the Rényi entropy $H_{i}$ associated with each self-similar partition. The performance analysis of the harmonic gasket antenna will be reported and discussed in greater detail in a forthcoming publication.

In summary, whenever the self-affine fractal antenna can be partitioned into self-similar pre-fractals, the entropy is given by Formula (10). In other cases, it is computed by the concept of multifractality. However, as in the case of fractal antennas, the value of such entropy describes the performance of the self-affine fractal antenna.

## 5. Conclusions

Harmonic analysis on fractal sets allows us to build a suitable generalization of the Sierpinski gasket *K* by the homeomorphism theory. More precisely, it is the homeomorphic image of *K*, called the harmonic Sierpinski gasket and denoted by $K_{H}$. Homeomorphisms preserve the topological structure. Hence, $K_{H}$ inherits some properties of *K*. In particular, Theorem 1 asserts that it is a self-affine fractal set. The self-affine fractal structure entails that it can be used as a geometric configuration in antenna design. The entropy of this antenna can be numerically computed by the concept of multifractality. In addition, the application described here shows that the numerical method given in [1] holds also for self-affine fractal antennas. The self-affine fractal structure provides flexibility in small antenna design. Therefore, the harmonic gasket antenna exhibits multiband resonance by selecting the proper scaling factor and optimization of the feed position. Further research should be done to generalize the definition of entropy for small antennas. Moreover, the analysis carried out could leads to an investigation of how the chaoticness of the geometric configuration influences the electromagnetic performance.

## Abbreviations

The following abbreviations are used in this manuscript:

HCR	Hohlfeld–Cohen–Rumsey
IFS	Iterated function system
*K*	Sierpinski gasket
$K_{H}$	Harmonic Sierpinski gasket
RF MEMS	Radio frequency microelectromechanical system
UMTS	Universal mobile telecommunications system
WLAN	Wireless local area network
